# Inequality of opportunity in a land of equal opportunities: The impact of parents’ health and wealth on their offspring’s quality of life in Norway

**DOI:** 10.1186/s12889-022-14084-x

**Published:** 2022-09-06

**Authors:** Espen Berthung, Nils Gutacker, Birgit Abelsen, Jan Abel Olsen

**Affiliations:** 1 Department of Community Medicine, Faculty of Health Sciences, UIT The Arctic University of Norway, Tromsø, Norway; 2 Centre of Health Economics, University of York, York, United Kingdom

**Keywords:** Inequality of opportunity, Childhood circumstances, Intergenerational transmission of health, EQ-5D, Abbrevations, IOp: Inequality of Opportunity, HRQoL: Health-Related Quality of Life, CFC: Childhood Financial Conditions, ITH: Intergenerational Transmission of Health, MO: Mobility, SC: Self-Care, UA: Usual Activities, PD: Pain & Discomfort, AX: Anxiety & Depression, GDP: Gross Domestic Product

## Abstract

**Background:**

The literature on Inequality of opportunity (IOp) in health distinguishes between *circumstances* that lie outside of own control vs*. efforts* that – to varying extents – are within one’s control. From the perspective of IOp, this paper aims to explain variations in individuals’ health-related quality of life (HRQoL) by focusing on two separate sets of variables that clearly lie outside of own control: Parents’ *health* is measured by their experience of somatic diseases, psychological problems and any substance abuse, while parents’ *wealth* is indicated by childhood financial conditions (CFC).

We further include own educational attainment which may represent a circumstance, *or* an effort, and examine associations of IOp for different health outcomes. HRQoL are measured by EQ-5D-5L utility scores, as well as the probability of reporting limitations on specific HRQoL-dimensions (mobility, self-care, usual-activities, pain & discomfort, and anxiety and depression).

**Method:**

We use unique survey data (*N* = 20,150) from the egalitarian country of Norway to investigate if differences in circumstances produce unfair inequalities in health. We estimate cross-sectional regression models which include age and sex as covariates. We estimate two model specifications. The first represents a narrow IOp by estimating the contributions of parents’ health and wealth on HRQoL, while the second includes own education and thus represents a broader IOp, alternatively it provides a comparison of the relative contributions of an effort variable and the two sets of circumstance variables.

**Results:**

We find strong associations between the circumstance variables and HRQoL. A more detailed examination showed particularly strong associations between parental psychological problems and respondents’ anxiety and depression. Our Shapley decomposition analysis suggests that parents’ health and wealth are each as important as own educational attainment for explaining inequalities in adult HRQoL.

**Conclusion:**

We provide evidence for the presence of the lasting effect of early life circumstances on adult health that persists even in one of the most egalitarian countries in the world. This suggests that there may be an upper limit to how much a generous welfare state can contribute to equal opportunities.

**Supplementary Information:**

The online version contains supplementary material available at 10.1186/s12889-022-14084-x.

## Background

Inequalities in health among socioeconomic groups are well documented in many countries and constitute a major policy concern. In her seminal paper, Whitehead held that for an inequality to be considered unfair “the *cause* has to be examined and *judged* to be unfair” [1]. Inspired by the conceptual dichotomy of *circumstances vs. efforts* [2, 3] an expanding literature in economics investigates the extent to which observed inequalities in health are caused by inequalities of opportunity (IOp) [4–8]. Circumstances are factors that lie *outside* of individuals’ control and, thus, something they cannot be held responsible for. If health inequalities are caused by systematic differences in circumstances, i.e. unequal opportunities, they are judged to be unfair. Efforts, on the other hand, reflect factors that are *within* individuals’ control and resulting inequalities are, therefore, not judged to be unfair [2, 9, 10]. The IOp literature distinguishes between *two* approaches: the *ex-ante* approach analyses IOp without considering effort, while *ex-post* analyses IOp when both circumstances and effort variables are considered [11, 12]. In the current paper, we adopt an *ex-ante* approach, followed by a model specification that includes a variable that can either be considered an additional circumstance, alternatively an effort.

This paper makes several contributions to the literature on IOp in health: First, except for Rivera [13], previous studies have either relied on ordinal, single-item measures of self-assessed health or have focused on narrowly defined aspects of health such as the presence of psychiatric disorders. These approaches fail to capture the multidimensional nature of health and how it affects different aspects of health-related quality of life (HRQoL). In this paper, health is measured by preference-based values obtained via the EQ-5D-5L instrument. Furthermore, we examine inequalities on opportunity with respect to different HRQoL dimensions (mobility, self-care, usual-activities, pain & discomfort, and anxiety & depression), which previous work has not explored. Second, we investigate the extent to which two different types of circumstances that both lie outside of individuals’ own control contribute to explaining inequalities in adult health. By considering childhood financial conditions, we contribute to a growing literature on the importance of childhood circumstances in determining adult health [14–17], particularly the financial environment in which children grow up [18–20]. Aside from the financial conditions during childhood, parents are likely to contribute to their offspring’s adult health by passing on some of their health stock (e.g. through genetics) and health-related behaviors [4, 21]. The existence of such *intergenerational transmission of health* (ITH) is well established. However, we extend this literature by the use of a comprehensive measure of parental health, i.e. the somatic *and* mental health of fathers *and* mothers. Beyond parents’ wealth and health, we consider the influence of own educational attainment. We take no position as to whether own education should be considered a circumstance [22] or effort [5]. Following on from this, we contribute to the literature by comparing the relative importance of childhood financial conditions (CFC), parental health and own education for explaining health inequalities. Our institutional context for studying inequality of opportunity in health is a country widely considered to be one of the most egalitarian in the world, with excellent access to public education, health care, and social security systems. At data collection, Norway was ranked 1st on the human development index compiled by the United Nations Development [23]. In addition, compared to other European countries, Norway have one of the lowest IOp for disposable income [24, 25]. Hence, Norway offers a useful 'best-case’ benchmark against which other countries can be compared.

## Methods

### Data sources

We used data from a large general population survey (conducted in 2015/16) of 21,083 individuals aged 40–97 years living in Tromsø, Norway. The study population is considered broadly representative of the Norwegian population aged 40 and above, however, with individuals holding a university degree being slightly overrepresented. The design of this Tromsø Study is described elsewhere [26].

### Health outcome

HRQoL was measured through the EQ-5D-5L instrument, in which respondents were asked to describe the level of problems they experience (either *no*, *slight*, *moderate*, *severe* or *extreme*) along five dimensions (mobility (denoted as MO), self-care (SC), usual activities (UA), pain and discomfort (PD), anxiety and depression (AD)) [27]. In the absence of a Norwegian value set, EQ-5D-5L responses were converted into utility scores using an amalgam value set of four Western countries [28]. To examine inequalities in the specific HRQoL domains, we dichotomize responses into *no problems* vs *any problems*, because in four of the five dimensions there were relatively few individuals reporting problems of any degree (see Table A1).

**Table 1:** Descriptive statistics of study sample

			EQ-5D-5L utility score	
	N	%	Mean	(SD)
**Total**	20,150	100%	0.890	(0.109)
**Sex**				
Women	10,558	52.4%	0.879	(0.114)
Men	9,592	47.6%	0.902	(0.102)
**Age**				
40–69 years	16,984	84.3%	0.892	(0.106)
70–79 years	2,508	12.4%	0.891	(0.113)
80 + years	658	3.3%	0.849	(0.146)
**Educational attainment**				
Primary school (10 years)	4,481	22.6%	0.873	(0.120)
Upper secondary school	5,509	27.8%	0.885	(0.108)
Lower university degree < 4 years	3,880	19.6%	0.895	(0.104)
Higher university degree ≥ 4 years	5,951	30.0%	0.906	(0.100)
**Childhood financial conditions (CFC)**				
Difficult	5,084	25.5%	0.869	(0.120)
Good	13,720	68.8%	0.897	(0.103)
Very Good	1,138	5.7%	0.907	(0.107)
**Parental health Number of somatic diseases**				
*Father*				
0	12,017	59.6%	0.894	(0.107)
1	5,656	28.1%	0.888	(0.109)
2 +	2,477	12.3%	0.879	(0.114)
*Mother*				
0	13,742	68.2%	0.894	(0.108)
1	4,812	23.9%	0.886	(0.109)
2 +	1,596	7.9%	0.870	(0.117)
**Psychological problem**				
*Father*				
No	19,396	96.3%	0.891	(0.108)
Yes	754	3.7%	0.862	(0.117)
*Mother*				
No	18,521	91.9%	0.893	(0.107)
Yes	1,629	8.1%	0.860	(0.127)
**Substance abuse**				
*Father*				
No	18,954	94.1%	0.891	(0.108)
Yes	1,196	5.9%	0.873	(0.119)
*Mother*				
No	19,814	98.3%	0.891	(0.108)
Yes	336	1.7%	0.857	(0.131)

### Explanatory variables

#### Parental health

Parents’ HRQoL was not assessed as part of the survey. Instead, respondents answered seven questions about their parents’ morbidity profiles on the day of the survey. Five questions (whether parents had been diagnosed with chest pain, stroke, asthma, diabetes, or had a heart attack before age 60) were used to calculate the total burden of somatic diseases (coded as 0, 1, or ≥ 2). As few respondents reported more than two chronic conditions, we chose a widely used measure of multimorbidity (MM2 +) as the top category [29]. Respondents were also asked whether their parents’ had known psychological problems and whether parents had had a history of alcohol and/or substance abuse. 

#### Childhood financial conditions

Childhood financial conditions (CFC) was measured by the question: ‘How was your family’s financial situation during your childhood?’ The response categories were: very good, good, difficult, and very difficult. The latter two categories were collapsed due to low frequency. 

#### Education level

Respondents’ level of educational attainment is categorized in line with the International Standard Classification of Education (ISCED): primary school (10 years); upper secondary school; lower university degree (< 4 years), and; higher university degree (≥ 4 years). 

#### Econometric specifications

We estimate the following cross-sectional regression model:${y}_{i}=f(\alpha +{{X}_{i}}^{^{\prime}}\beta )+ {\varepsilon }_{i}$. Here, ${y}_{i}$ is a measure of HRQoL for individual$i=1,\dots ,N$, ${X}_{i}$ is a matrix of explanatory variables, *f* is a link function and ${\varepsilon }_{i}$ is the error term. We estimate two specifications, with and without the inclusion of own education. We also provide three partial regression models for each set of the explanatory variables. Thereby, we can compare the coefficients’ standard errors and magnitude in the partial models with those in the full model, and thus identify the extent of multicollinearity. All models include age and sex as covariates. Age was coded in three bands: 40–69, 70–79, and 80 + . The larger age band 40–69 was chosen because previous analysis showed that HRQoL is approximately stable until the late sixties before it declines [30].

Model specification 1 includes CFC and parental health, both of which reflect circumstances outside of own control. Model 2 further includes respondents’ highest educational attainment. To account for heterogeneity across sexes [31], this main model was also estimated separately for men (Model 2M) and women (Model 2W). We quantify the relative importance of *each* explanatory variable for the overall R^2^ by using the Shapley decomposition method. This decomposition derives the marginal effect of the explanatory variables on the R^2^ by eliminating each variable in sequence, and then assigns to each variable the average of its marginal contributions in all possible elimination sequences [32, 33].

Finally, by comparing the magnitude of the education coefficients in the partial Model Edu (Table A2) with those in the full Model 2, we get an indication of the extent to which the associations between own education and HRQoL operates through parent’s health and wealth.

**Table 2:** Linear regression on the EQ-5D-5L utility score

	Full sample		Men	Women
Variables	Model 1	Model 2	Model 2M	Model 2W
Intercept	0.896***	0.879***	0.898***	0.884***
	(0.001)	(0.002)	(0.003)	(0.003)
Men	0.021***	0.022***		
	(0.002)	(0.002)		
**Age groups (Ref. 40–69)**				
70–79	0.000	0.005**	0.012***	-0.002
	(0.002)	(0.002)	(0.003)	(0.004)
80 +	-0.045***	-0.036***	-0.022***	-0.048***
	(0.004)	(0.005)	(0.006)	(0.007)
**Childhood financial conditions (Ref. Good)**				
Difficult	-0.026***	-0.024***	-0.020***	-0.027***
	(0.002)	(0.002)	(0.002)	(0.003)
Very good	0.010***	0.008**	0.006	0.010**
	(0.003)	(0.003)	(0.005)	(0.005)
**Number of somatic diseases (Ref. 0)**				
Father 1	-0.004**	-0.004**	-0.004*	-0.004
	(0.002)	(0.002)	(0.002)	(0.002)
Father 2 +	-0.012***	-0.012***	-0.012***	-0.012***
	(0.002)	(0.002)	(0.003)	(0.003)
Mother 1	-0.004**	-0.003	-0.002	-0.003
	(0.002)	(0.002)	(0.003)	(0.003)
Mother 2 +	-0.016***	-0.014***	-0.009**	-0.017***
	(0.003)	(0.003)	(0.004)	(0.004)
**Psychological problem (Ref. No)**				
Father: Yes	-0.020***	-0.022***	-0.024***	-0.020***
	(0.004)	(0.004)	(0.006)	(0.005)
Mother: Yes	-0.025***	-0.027***	-0.020***	-0.032***
	(0.003)	(0.003)	(0.004)	(0.004)
**Substance abuse (Ref. No)**				
Father: Yes	-0.009***	-0.010***	-0.010**	-0.010**
	(0.003)	(0.003)	(0.004)	(0.005)
Mother: Yes	-0.016***	-0.018***	-0.021**	-0.015*
	(0.006)	(0.006)	(0.009)	(0.008)
**Educational attainment (Ref. Primary school 10 years)**				
Upper secondary school		0.008***	0.011***	0.004
		(0.002)	(0.003)	(0.003)
Lower university degree < 4 years		0.019***	0.020***	0.015***
		(0.002)	(0.003)	(0.004)
Higher university degree ≥ 4 years		0.030***	0.027***	0.030***
		(0.002)	(0.003)	(0.003)
R^2^	0.041	0.051	0.031	0.050

Note: *p < 0.1, **p < 0.05, ***p < 0.01

All models were estimated by OLS (utility scores) or logit regressions (dimension responses). We do not model responses on the EQ-dimensions as ordered outcomes, because few individuals report worse levels than *slight* problems (see Table A1), and because the proportional odds assumption was found to be violated in our data. To explore potential cohort effects, we also estimated separated regressions (based on Model 2) for individuals aged 40–49; 50–59; 60–69, and 70 + .

In the sensitivity analyse*s,* we first wanted to assess the appropriateness of the main model specification. For this, we apply the least absolute shrinkage and selection operator (LASSO) method. The LASSO method standardizes predictors and utilizes a regularization factor, the L1-norm or lambda (λ), to maximize the out-of-sample model fit by applying a penalty to predictor coefficients. This removes predictors that do not contribute to the out-of-sample performance of the model [34]. In the next sensitivity analysis, we split the sample into four based on the age bands (40–49; 50–59; 60–69, and 70 + .) and rerun the main specification on these subsamples.

All analyses were conducted using R version 1.4.1106; packages used were stats, relaimpo, margins, glmnet, and caret.

## Results

### Main results

Table 1 provides descriptive statistics of the sample and mean utility scores by level of respondent characteristic. Table 2 presents the main regression results by use of two model specifications, and with EQ-5D-5L utility scores as dependent variable. The stable standard errors and coefficients across the two models indicate that the key sets of predictors are independent of each other. Furthermore, by comparing the standard errors and coefficients in the three partial model specifications (Table A2) with those in the full Model 2, there is further evidence that multicollinearity is not a problem; i.e. each of our three sets of predictors are independent of each other. Note in particular that the education coefficients and their standard errors in Model 2 are remarkably similar to those in the partial model (Table A2).

Now, we focus on results from Model 2. The difference in adult HRQoL between having had Very good vs. Difficult CFC (0.008 – (-0.024) = 0.032) is approximately equal to the education gap (= 0.030). All three measures of parental health have statistically significant effects on respondents’ adult HRQoL. In Model 2M and 2W, there are some noteworthy differences between men and women: difficult CFC and mothers’ somatic diseases and psychological problem affect women more than men.

Table 3 provides the coefficient estimates from the logit regression models and the average marginal effect of variables on the probability of reporting *no problems*, for each EQ-dimension. There is considerable heterogeneity across dimensions. For example, having experienced difficult CFC reduces the probability of reporting no problems with Pain/discomfort by -6.9 percentage points (pp) compared to -1.7 pp for Self-care. Parental psychological problems affect own Anxiety/depression most, whereas parental somatic problems are most closely associated with Pain/discomfort, Mobility and Usual activities.

**Table 3:** Logistic regression results of the probability of reporting no problems on the EQ-5D-5L dimensions

	Mobility		Self-care		Usual activities		Pain discomfort		Anxiety/depression		
	Est(SE)	AME(SE)	Est(SE)	AME(SE)	Est(SE)	AME(SE)	Est(SE)	AME(SE)	Est(SE)	AME(SE)	
Intercept	1.462***		3.323***		1.327***		-0.908***		1.186***		
	(0.052)		(0.102)		(0.052)		(0.045)		(0.049)		
Men	0.337***	0.046***	0.155**	0.006**	0.638***	0.085***	0.384***	0.083***	0.288***	0.048***	
	(0.039)	(0.005)	(0.074)	(0.003)	(0.041)	(0.005)	(0.031)	(0.007)	(0.036)	(0.006)	
**Age groups (ref. 40–69)**											
70–79	-0.357***	-0.052***	-0.198*	-0.008*	0.098	0.013	0.187***	0.041***	0.495***	0.074***	
	(0.056)	(0.009)	(0.108)	(0.005)	(0.062)	(0.008)	(0.048)	(0.011)	(0.062)	(0.008)	
80 +	-1.371***	-0.258***	-0.954***	-0.054***	-0.552***	-0.087***	0.198**	0.044**	0.229**	0.037**	
	(0.090)	(0.021)	(0.153)	(0.012)	(0.100)	(0.018)	(0.092)	(0.021)	(0.111)	(0.017)	
**Childhood financial conditions (Ref = Good)**											
Difficult	-0.340***	-0.049***	-0.405***	-0.017***	-0.374***	-0.053***	-0.330***	-0.069***	-0.350***	-0.061***	
	(0.043)	(0.006)	(0.079)	(0.004)	(0.043)	(0.006)	(0.038)	(0.008)	(0.039)	(0.007)	
Very Good	-0.065	-0.009	0.056	0.002	0.154	0.019*	0.250***	0.057***	0.401***	0.057***	
	(0.087)	(0.012)	(0.176)	(0.006)	(0.093)	(0.011)	(0.064)	(0.015)	(0.088)	(0.011)	
**Number of somatic diseases (Ref. 0)**											
Father 1	-0.048	-0.006	-0.097	-0.004	-0.086*	-0.011*	-0.090**	-0.020**	-0.031		-0.005
	(0.044)	(0.006)	(0.083)	(0.003)	(0.044)	(0.006)	(0.035)	(0.008)	(0.040)		(0.007)
Father 2 +	-0.192***	-0.027***	-0.251**	-0.010**	-0.193***	-0.027***	-0.281***	-0.059***	-0.103*		-0.017*
	(0.058)	(0.009)	(0.107)	(0.005)	(0.059)	(0.008)	(0.050)	(0.010)	(0.054)		(0.009)
Mother 1	-0.150***	-0.021***	-0.084	-0.003	-0.062	-0.008	-0.075**	-0.016**	0.030		0.005
	(0.045)	(0.006)	(0.086)	(0.003)	(0.046)	(0.006)	(0.037)	(0.008)	(0.042)		(0.007)
Mother 2 +	-0.378***	-0.056***	-0.228*	-0.009*	-0.315***	-0.045***	-0.344***	-0.071***	0.012		0.002
	(0.066)	(0.011)	(0.125)	(0.006)	(0.066)	(0.010)	(0.063)	(0.012)	(0.065)		(0.011)
**Psychological problem (Ref. No)**											
Father: Yes	0.023	0.003	-0.192	-0.008	-0.232**	-0.033**	-0.145*	-0.031*	-0.885***		-0.177***
	(0.103)	(0.014)	(0.179)	(0.008)	(0.096)	(0.015)	(0.085)	(0.018)	(0.079)		(0.018)
Mother: Yes	-0.106	-0.015	-0.199	-0.008	-0.311***	-0.045***	-0.222***	-0.046***	-0.754***		-0.146***
**Substance abuse (Ref. No)**	(0.070)	(0.010)	(0.126)	(0.006)	(0.067)	(0.010)	(0.061)	(0.012)	(0.057)		(0.012)
Father: Yes	-0.146*	-0.021*	0.024	0.001	0.022	0.003	-0.065	-0.014	-0.231***		-0.040***
	(0.080)	(0.012)	(0.155)	(0.006)	(0.083)	(0.011)	(0.068)	(0.014)	(0.069)		(0.013)
Mother: Yes	-0.421***	-0.065***	-0.123	-0.005	-0.208	-0.030	-0.164	-0.034	-0.099		-0.017
	(0.136)	(0.023)	(0.268)	(0.011)	(0.140)	(0.021)	(0.131)	(0.027)	(0.125)		(0.022)
**Educational attainment (Ref. Primary school 10 years)**											
Upper secondary school	0.159***	0.025***	-0.075	-0.003	0.069	0.011	-0.002	0.000	0.092*		0.016*
	(0.051)	(0.008)	(0.098)	(0.004)	(0.052)	(0.008)	(0.046)	(0.009)	(0.050)		(0.009)
Lower university degree < 4 years	0.374***	0.055***	0.093	0.004	0.304***	0.044***	0.234***	0.050***	0.097*		0.017*
	(0.059)	(0.009)	(0.112)	(0.004)	(0.060)	(0.009)	(0.049)	(0.010)	(0.055)		(0.009)
Higher university degree ≥ 4 years	0.643***	0.088***	0.382***	0.013***	0.634***	0.083***	0.455***	0.100***	0.217***		0.036***
	(0.056)	(0.008)	(0.109)	(0.004)	(0.057)	(0.008)	(0.045)	(0.010)	(0.051)		(0.008)
Pseudo R2: McFadden	0.041		0.018		0.037		0.023		0.033		

Note: *p < 0.1, **p < 0.05, ***p < 0.01. *Est* Estimated coefficient on logit scale, *AME* Average marginal effect on probability scale, *SE* Standard error

**Fig. 1:**
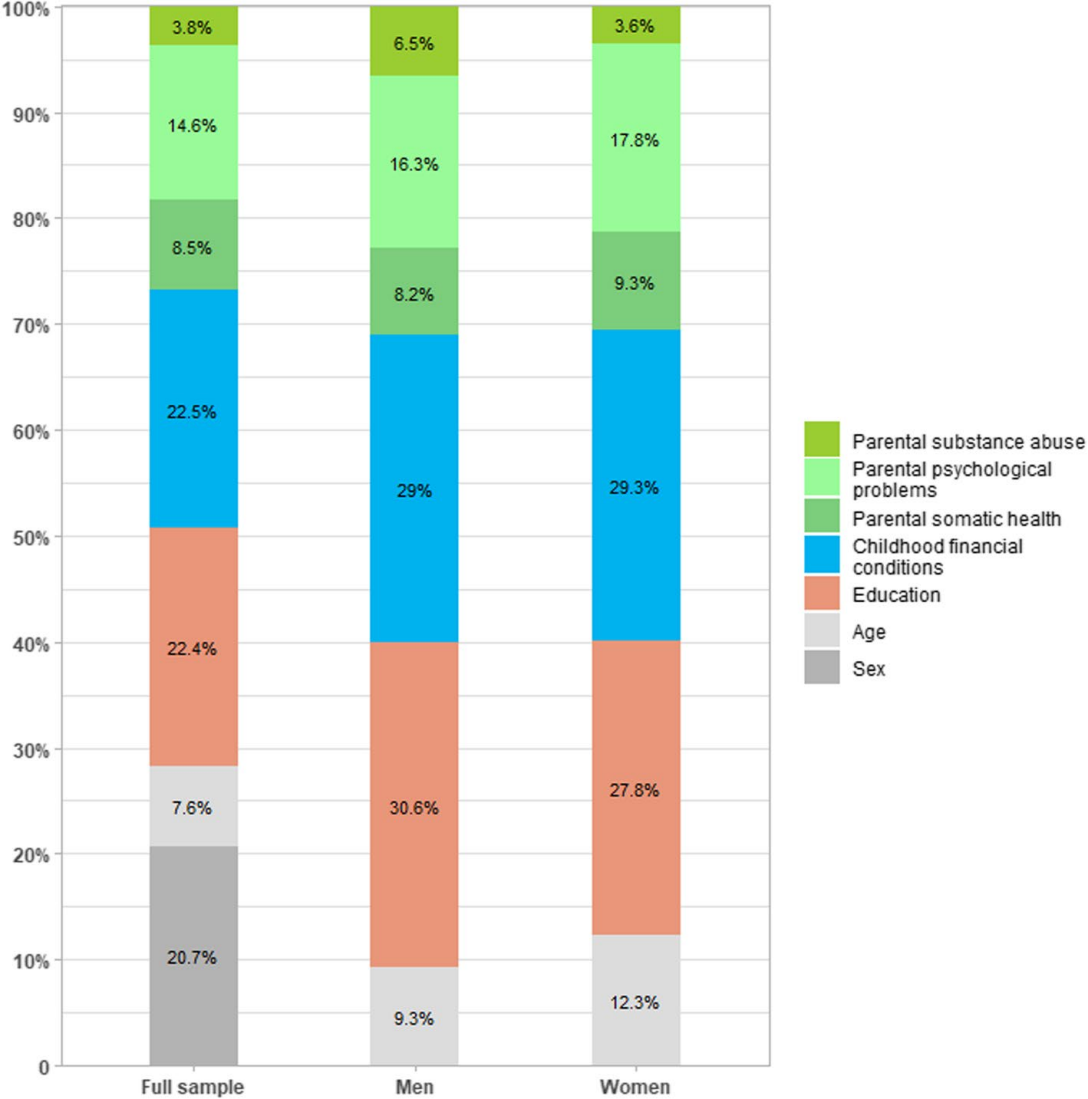
Shapley decomposition of explained variance (R^2^ for utility score) based on model specification 2, 2M, and 2W

The Shapley decomposition analyses in Fig. 1 illustrate the relative importance of CFC, parental health, and own educational attainment for respondents’ HRQoL for the pooled sample and separately for each sex. In the pooled sample analysis, CFC and parental health account for nearly 50% of the explained variance, while educational attainment account for 22.4%. For both sexes, the relative importance of the three main predictors appear broadly similar: parental health variables together explain around 31%; CFC slightly less (29%), while own education is relatively more important in explaining men’s HRQoL.

### Sensitivity analysis

For the LASSO method, we choose the optimal parameterization of lambda by means of 10-fold cross validation. After regularizing the model, all parameters were non-zero, thus supporting the appropriateness of the model specification.

Table A3 shows results by age groups. The effects of parents’ psychological problems and substance abuse are more pronounced in younger respondents, which may reflect cohort differences in the awareness of mental health and substance abuse. For example, the oldest cohort reported much lower frequencies of parents’ mental health problems (Table A4). The HRQoL-gap due to CFC is larger in the oldest age group, suggesting life-long effects of CFC. The educational gradient is more pronounced in younger respondents but diminishes around retirement age.

## Discussion

This study contributes to the growing literature on inequalities in opportunity by providing new evidence from one of the wealthiest and most equal countries in the world on the extent that circumstances such as parental health and CFC have lasting impacts on adult HRQoL. Earlier Norwegian studies on IOp have focused on child-care [35], education [36] and income [37]. However, we have not identified Norwegian IOp-studies on health that have included parental health. Our results show parents’ somatic health affect their offspring’s pain and functional ability, while parents’ psychological problems and substance abuse have substantial effects on their children’s self-reported levels of anxiety/depression.

Furthermore, our findings support previous studies from other countries which show lasting impacts of CFC on adult health [19], and we find these to have similar magnitude to the impact of educational attainment. Interestingly, the distributions of respondents on the three CFC-levels are remarkably similar across age-cohorts (Table A4), whose *absolute* standard of living during childhood increased tremendously over time (approximately 3% p.a. GDP/capita growth between 1950 and 1990). This suggest that our measure of CFC represents a good proxy for *relative* deprivation. Finally, the Shapley analysis showed that CFC and parental health are each as important for HRQoL as own educational attainment.

We found evidence of heterogeneity by sex in how much circumstances affect descendants’ health. As for parental health, the general pattern is that fathers’ ill health have similar effects on sons and daughters, while mothers’ ill health have stronger effects on daughters. However, sons appear to be relatively more negatively affected than daughters by their fathers’ substance abuse and psychological problems. As for the ‘social lottery’ of early life, childhood financial conditions appear to be more important for women’s than men’s adult health.

While CFC and parental health are assumed to reflect circumstances, own educational attainment is arguably *partly* outside of one’s control and therefore more difficult to locate on the circumstances-efforts continuum. Previous work has considered education either as circumstance [22] or effort [5]. This disagreement in the literature emphasizes the importance of defining an *age of consent* to delineate circumstances from effort as suggested by Arneson [38] and empirically investigated by Hufe [39]. In this paper, we prefer to take no firm position on this issue. However, we do observe that the estimated effect of educational attainment on HRQoL is remarkably stable across econometric specifications, indicating that it is largely independent of assumed circumstances (i.e. CFC and parental health).

We acknowledge that our categorization of parental health as *circumstances* might be suggestive of inherited genetics that are outside of children’s control. However, parents’ ill health may have been caused in part by their health-related behaviors or unhealthy habits, which they can pass on to their children (e.g. Balasooriya [40]). While it certainly takes efforts to quit inherited bad habits, they may be easier to alter than bad genes. Thus, focusing on unhealthy habits may appeal to policymakers who seek to tackle health inequalities in their communities.

Our study has some limitations. First, we approximate parents’ health through their morbidities burden sometime *after* their offspring are likely to have left the nest. We are therefore cautious in interpreting these results to reflect any particular pathway of intergenerational transmission of health (i.e. genetics, habits). Second, parents’ morbidity patterns and health-related behaviors are likely to be incomplete proxies of the parental health stock and its determinants. Finally, we cannot rule out reverse causality in which children’s poor health requires parents to take on care duties, with negative consequences for parental health.

In this paper, we have focused on two sets of circumstance variables that are clearly outside of own control, and further included one variable, education, that lies somewhere in between the end points on the *circumstances-effort continuum*. Certainly, there is a need for research that includes more variables that lie towards the effort-end on this continuum, i.e. indicators of health related behaviour, e.g. physical activity. Such research would provide important knowledge on the difficult question: how much of observed health inequalities reflect inequalities in opportunity, and hence considered *unfair*, as compared to how much that reflect own choices, and hence considered *acceptable*?

We have shown that even in a land of equal opportunities, large inequalities in HRQoL are caused by circumstances beyond individuals’ control. If Norway cannot eradicate unfair inequalities in health, other countries will also struggle. This suggests that there may be an upper limit to how much a generous welfare state can contribute to equal opportunities.

## Supplementary Information

**Additional file 1:** **Table A1.** Distributions of EQ-5D-5L responses by dimension (N, %). **Table A2. **Linear regressions on the EQ-5D-5L utility score. Partial effects: parents’ wealth (Model PW); parents’ health (Model PH); own education (Model Edu).** Table A3.** Analysis of utility scores by age-groups (Model 2 specification).** Table A4.** Descriptive statistics (N, %) by age groups.

## Data Availability

Since the data contains potentially identifying or sensitive information about the participants in the Tromsø study, we are not allowed to share a data set. Contact information for the Tromsø study can be found by the following link: https://uit.no/research/ tromsostudy/project?pid = 709,148.

## References

[1] Whitehead M. The concepts and principles of equity and health. Int J Health Serv. 1992;22(3):429–45 (p. 431). 10.2190/986L-LHQ6-2VTE-YRRN 1644507

[2] Roemer JE. Equality of opportunity: A progress report. Soc Choice Welf. 2002;19(2):455–71.

[3] Roemer, J.E. Theories of distributive justice. Cambridge: Harvard University Press; 1998.

[4] Trannoy A, et al. Inequality of opportunities in health in France: a first pass. Health Econ. 2010;19(8):921–38. 10.1002/hec.1528 19588460

[5] Rosa Dias P. Inequality of opportunity in health: evidence from a UK cohort study. Health Econ. 2009;18(9):1057–74. 10.1002/hec.1535 19644964

[6] Rosa Dias P. Modelling opportunity in health under partial observability of circumstances. Health Econ. 2010;19(3):252–64. 10.1002/hec.1584 20049898

[7] Fajardo-Gonzalez J (2016). Inequality of opportunity in adult health in Colombia. J Econ Inequality.

[8] Deutsch J, Alperin MNP, Silber J (2018). Using the Shapley decomposition to disentangle the impact of circumstances and efforts on health inequality. Soc Indic Res.

[9] Cappelen AW, Norheim OF (2005). Responsibility in health care: a liberal egalitarian approach. J Med Ethics.

[10] Olsen JA. Concepts of equity and fairness in health and health care, in he Oxford Handbook of Health Economics. UK: Oxford University Press; 2011.

[11] Ferreira FH, Gignoux J (2011). The measurement of inequality of opportunity: Theory and an application to Latin America. Rev Income Wealth.

[12] Checchi D, Peragine V (2010). Inequality of opportunity in Italy. J Econ Inequality.

[13] Rivera F (2017). Health opportunities in Colombia. Lecturas de Economía.

[14] Case A, Fertig A, Paxson C (2005). The lasting impact of childhood health and circumstance. J Health Econ.

[15] Marmot M (2001). Relative contribution of early life and adult socioeconomic factors to adult morbidity in the Whitehall II study. J Epidemiol Community Health.

[16] Galobardes B, Lynch JW, Davey Smith G (2004). Childhood socioeconomic circumstances and cause-specific mortality in adulthood systematic review and interpretation. Epidemiol Rev.

[17] Veenstra G, Vanzella-Yang A. Interactions between parental and personal socioeconomic resources and self-rated health: Adjudicating between the resource substitution and resource multiplication theories. Soc Sci Med. 2022;292:114565. 10.1016/j.socscimed.2021.114565 34801333

[18] Widding-Havneraas T, Pedersen SH (2020). The role of welfare regimes in the relationship between childhood economic stress and adult health: A multilevel study of 20 European countries. SSM-Popul Health.

[19] Case A, Lubotsky D, Paxson C (2002). Economic status and health in childhood: The origins of the gradient. Ame Econ Rev.

[20] Clark AE, d'Ambrosio C, Barazzetta M (2021). Childhood circumstances and young adulthood outcomes: The role of mothers' financial problems. Health Econ.

[21] Schulkind L (2017). Getting a sporting chance: Title IX and the intergenerational transmission of health. Health Econ.

[22] Davillas A, Jones AM (2020). Ex ante inequality of opportunity in health, decomposition and distributional analysis of biomarkers. J Health Econ.

[23] Jahan S. Human development report 2015. Vol. 25. United Nations Development Programme (UNDP). 2015. p. 274.

[24] Checchi D, Peragine V, Serlenga L. Inequality of Opportunity in Europe: Is There a Role for Institutions? In Cappellari, L., Polachek, S., and Tatsiramos, K., editors, Inequality: Causes and Consequences, volume 43 of Research in Labor Economics. Bingley: Emerald; 2016. p. 1–44.

[25] Suárez Álvarez A, Lopez Menendez AJ (2021). Dynamics of inequality and opportunities within European countries. Bull Econ Res.

[26] Jacobsen BK (2011). Cohort profile: the Tromsø study. Int J Epidemiol.

[27] Herdman M (2011). Development and preliminary testing of the new five-level version of EQ-5D (EQ-5D-5L). Qual Life Res.

[28] Olsen JA, Lamu AN, Cairns J (2018). In search of a common currency: A comparison of seven EQ-5D-5L value sets. Health Econ.

[29] Chua YP (2021). Definitions and prevalence of multimorbidity in large database studies: A scoping review. Int J Environ Res Pub Health.

[30] Olsen JA, Lindberg MH, Lamu AN. Health and wellbeing in Norway: Population norms and the social gradient. Soc Sci Med. 2020;259:113155. 10.1016/j.socscimed.2020.113155 32650252

[31] Janssen B, Szende A. Population norms for the EQ-5D. Self-Reported Population Health: An International Perspective Based on EQ-5D. 2014. p. 19–30. 29787044

[32] Israeli O (2007). A Shapley-based decomposition of the R-square of a linear regression. J Econo Inequality.

[33] Shorrocks AF (2013). Decomposition procedures for distributional analysis: a unified framework based on the Shapley value. J Econ Inequality.

[34] Tibshirani R (1996). Regression shrinkage and selection via the lasso. J Roy Stat Soc: Ser B (Methodol).

[35] Drange N, Telle K. Universal child care and inequality of opportunity. Descriptive findings from Norway. Discussion Papers, 879, Research Department of Statistics Norway . 2018. p. 1–43

[36] Reisel L (2011). Two paths to inequality in educational outcomes: Family background and educational selection in the United States and Norway. Sociol Educ.

[37] Aaberge R, Mogstad M, Peragine V (2011). Measuring long-term inequality of opportunity. J Public Econ.

[38] Arneson RJ (1990). Liberalism distributive subjectivism, and equal opportunity for welfare. Philos Pub Aff.

[39] Hufe P (2017). Inequality of income acquisition: the role of childhood circumstances. Soc Choice Welf.

[40] Balasooriya NN, Bandara JS, Rohde N (2021). The intergenerational effects of socioeconomic inequality on unhealthy bodyweight. Health Econ..

