# Supplementary material for: Inequality of opportunity in a land of equal opportunities: The impact of parents’ health and wealth on their offspring’s quality of life in Norway
Source: BMC Public Health. 2022 Sep 6;22:1691. doi: 10.1186/s12889-022-14084-x (PMC9450446; doi:10.1186/s12889-022-14084-x)
Supplement: Supplementary file 1 — Additional file 1: Table A1. Distributions of EQ-5D-5L responses by dimension (N, %). Table A2. Linear regressions on the EQ-5D-5L utility score. Partial effects: parents’ wealth (Model PW); parents’ health (Model PH); own education (Model Edu). Table A3. Analysis of utility scores by age-groups (Model 2 specification). Table A4. Descriptive statistics (N, %) by age groups. [file 12889_2022_14084_MOESM1_ESM.docx]

**Appendix**

| **Table A1:** Distributions of EQ-5D-5L responses by dimension (N, %) | | | | | |
| --- | --- | --- | --- | --- | --- |
|  | MO | SC | UA | PD | AD |
| No problems | 16,616 | 19,323 | 16,766 | 6,727 | 15,700 |
|  | (82.5%) | (96.1%) | (83.2%) | (33.4%) | (78.1%) |
| Slight problems | 2,638 | 701 | 2,569 | 9,952 | 3,574 |
|  | (13.1%) | (3.5%) | (12.7%) | (49.4%) | (17.6%) |
| Moderate problems | 619 | 83 | 591 | 2,720 | 705 |
|  | (3.1%) | (0.4%) | (2.9%) | (13.5%) | (3.5%) |
| Severe problems | 249 | 28 | 190 | 682 | 153 |
|  | (1.2%) | (0.1%) | (0.9%) | (3.4%) | (0.7%) |
| Unable/extreme problems | 28 | 15 | 34 | 69 | 18 |
|  | (0.1%) | (0.07%) | (0.2%) | (0.3%) | (0.1%) |
| Total | 20150 | 20150 | 20150 | 20150 | 20150 |
|  | (100%) | (100%) | (100%) | (100%) | (100%) |
| Note: MO, mobility; SC, self-care; UA, usual activities PD, pain/discomfort; AD, anxiety/depression | | | | | |

| **Table A2:** Linear regressions on the EQ-5D-5L utility score. Partial effects: parents’ wealth (Model PW); parents’ health (Model PH); own education (Model Edu) | | | | | | |
| --- | --- | --- | --- | --- | --- | --- |
| Variables |  | Model PW |  | Model PH |  | Model Edu |
| Intercept |  | 0.887*** |  | 0.892*** |  | 0.863*** |
|  |  | (0.001) |  | (0.001) |  | (0.002) |
| Men |  | 0.023*** |  | 0.021*** |  | 0.023*** |
|  |  | (0.002) |  | (0.001) |  | (0.001) |
| **Age groups (Ref. 40-69)** |  |  |  |  |  |  |
| 70-79 |  | 0.002 |  | -0.003 |  | 0.005** |
|  |  | (0.002) |  | (0.002) |  | (0.002) |
| 80+ |  | -0.041*** |  | -0.046*** |  | -0.031*** |
|  |  | (0.003) |  | (0.004) |  | (0.004) |
| **Childhood financial conditions (Ref. Good)** |  |  |  |  |  |  |
| Difficult |  | -0.029*** |  |  |  |  |
|  |  | (0.002) |  |  |  |  |
| Very good |  | 0.010*** |  |  |  |  |
|  |  | (0.003) |  |  |  |  |
| **Number of somatic diseases (Ref. 0)** |  |  |  |  |  |  |
| Father 1 |  |  |  | -0.004*** |  |  |
|  |  |  |  | (0.001) |  |  |
| Father 2+ |  |  |  | -0.012*** |  |  |
|  |  |  |  | (0.002) |  |  |
| Mother 1 |  |  |  | -0.005*** |  |  |
|  |  |  |  | (0.001) |  |  |
| Mother 2+ |  |  |  | -0.019*** |  |  |
|  |  |  |  | (0.002) |  |  |
| **Psychological problem (Ref. No)** |  |  |  |  |  |  |
| Father: Yes |  |  |  | -0.022*** |  |  |
|  |  |  |  | (0.004) |  |  |
| Mother: Yes |  |  |  | -0.027*** |  |  |
|  |  |  |  | (0.002) |  |  |
| **Substance abuse (Ref. No)** |  |  |  |  |  |  |
| Father: Yes |  |  |  | -0.013*** |  |  |
|  |  |  |  | (0.003) |  |  |
| Mother: Yes |  |  |  | -0.016*** |  |  |
|  |  |  |  | (0.006) |  |  |
| **Educational attainment (Ref. Primary school 10 years)** |  |  |  |  |  |  |
| Upper secondary school |  |  |  |  |  | 0.009*** |
|  |  |  |  |  |  | (0.002) |
| Lower university degree < 4 years |  |  |  |  |  | 0.019*** |
|  |  |  |  |  |  | (0.002) |
| Higher university degree ≥ 4 years |  |  |  |  |  | 0.032*** |
|  |  |  |  |  |  | (0.002) |
| R^2^ |  | 0.030 |  | 0.029 |  | 0.027 |
| Note: *p<0.1, **p<0.05, ***p<0.01 | | | | | | |

| **Table A3:** Analysis of utility scores by age-groups (Model 2 specification) | | | | | | | | | |
| --- | --- | --- | --- | --- | --- | --- | --- | --- | --- |
|  |  | Age: 40-49 |  | Age: 50-59 |  | Age: 60-69 |  | Age: 70+ | |
|  |  | N=6,214 |  | N=5,803 |  | N=4,967 |  | N=3,166 | |
| Intercept |  | 0.855*** |  | 0.869*** |  | 0.894*** |  | 0.872*** | |
|  |  | (0.005) |  | (0.004) |  | (0.004) |  | (0.005) | |
| Men |  | 0.023*** |  | 0.023*** |  | 0.015*** |  | 0.039*** | |
|  |  | (0.003) |  | (0.003) |  | (0.003) |  | (0.005) | |
| **Childhood financial conditions (Ref=Good)** | | |  |  |  |  |  |  | |
| Difficult |  | -0.026*** |  | -0.020*** |  | -0.021*** |  | -0.029*** | |
|  |  | (0.003) |  | (0.003) |  | (0.003) |  | (0.005) | |
| Very Good |  | 0.007 |  | 0.007 |  | 0.005 |  | 0.025** | |
|  |  | (0.006) |  | (0.006) |  | (0.006) |  | (0.010) | |
| **Number of somatic diseases (Ref. 0)** |  |  |  |  |  |  |  |  | |
| Father 1 |  | -0.008** |  | -0.003 |  | -0.004 |  | 0.001 | |
|  |  | (0.003) |  | (0.003) |  | (0.003) |  | (0.005) | |
| Father 2+ |  | -0.010** |  | -0.014*** |  | -0.012*** |  | -0.010 | |
|  |  | (0.004) |  | (0.004) |  | (0.004) |  | (0.008) | |
| Mother 1 |  | -0.004 |  | -0.003 |  | -0.001 |  | -0.005 | |
|  |  | (0.003) |  | (0.003) |  | (0.003) |  | (0.005) | |
| Mother 2+ |  | -0.017*** |  | -0.009* |  | -0.015*** |  | -0.018** | |
|  |  | (0.006) |  | (0.005) |  | (0.005) |  | (0.008) | |
| **Psychological problem (Ref. No)** |  |  |  |  |  |  |  |  | |
| Father: Yes |  | -0.027*** |  | -0.024*** |  | -0.013* |  | -0.006 | |
|  |  | (0.006) |  | (0.007) |  | (0.008) |  | (0.019) | |
| Mother: Yes |  | -0.029*** |  | -0.034*** |  | -0.018*** |  | -0.009 | |
|  |  | (0.005) |  | (0.005) |  | (0.006) |  | (0.011) | |
| **Substance abuse (Ref. No)** |  |  |  |  |  |  |  |  |  |
|  |  | -0.001 |  | -0.024*** |  | -0.010 |  | 0.015 | |
| Father: Yes |  | (0.005) |  | (0.006) |  | (0.007) |  | (0.013) | |
|  |  | -0.017** |  | -0.007 |  | -0.021 |  | -0.031 | |
| Mother: Yes |  | (0.008) |  | (0.012) |  | (0.014) |  | (0.033) | |
| **Educational attainment (Ref. Primary school 10 years)** | | |  |  |  |  |  |  | |
| Upper secondary school |  | 0.029*** |  | 0.014*** |  | 0.006* |  | 0.002 | |
|  |  | (0.005) |  | (0.004) |  | (0.004) |  | (0.006) | |
| Lower university degree < 4 years |  | 0.041*** |  | 0.028*** |  | 0.011*** |  | 0.012* | |
|  |  | (0.005) |  | (0.005) |  | (0.004) |  | (0.007) | |
| Higher university degree ≥ 4 years |  | 0.058*** |  | 0.040*** |  | 0.016*** |  | 0.011 | |
|  |  | (0.005) |  | (0.004) |  | (0.004) |  | (0.007) | |
| R2 |  | 0.070 |  | 0.056 |  | 0.032 |  | 0.050 | |
|  | | | | | | | | | |

| **Table A4** Descriptive statistics (N, %) by age groups | | | | |
| --- | --- | --- | --- | --- |
|  | 40-49 (N=6,214) | 50-59 (N=5,803) | 60-69 (N=4,967) | 70+ (N=3,166) |
| EQ-5D-5L utility score SD | 0.892 (0.108) | 0.888(0.110) | 0.896(0.100) | 0.882(0.122) |
| Sex |  |  |  |  |
| Women | 3,261 (52.5%) | 3,120 (53.8%) | 2,555 (51.4%) | 1,622 (51.2%) |
| Men | 2,953 (47.5%) | 2,683 (46.2%) | 2,412 (48.6%) | 1,544 (48.8%) |
|  | |  |  |  |
| **Educational attainment** | |  |  |  |
| Primary school (10 years) | 595 (9.6%) | 1,052 (18.3%) | 1,497 (30.6%) | 1,337 (44.7%) |
| Upper secondary school | 1,685 (27.3%) | 1,726 (30.0%) | 1,336 (27.3%) | 762 (25.5%) |
| Lower university degree <4 years | 1,352 (21.9%) | 1,213 (21.1%) | 870 (17.8%) | 445 (14.9%) |
| Higher university degree ≥4 years | 2,550 (41.2%) | 1,761 (30.6%) | 1,196 (24.4%) | 444 (14.9%) |
| **Childhood financial conditions (CFC)** | |  |  |  |
| Difficult | 1,320 (21.3%) | 1,306 (22.6%) | 1,391 (28.4%) | 1,067 (34.6%) |
| Good | 4,501 (72.8%) | 4,106 (71.1%) | 3,251 (66.3%) | 1,862 (60.4%) |
| Very Good | 365 (5.9%) | 360 (6.2%) | 260 (5.3%) | 153 (5.0%) |
| **Parental health**  **Number of somatic diseases** | |  |  |  |
| Father |  |  |  |  |
| 0 | 3,973 (63.9%) | 3,200 (55.1%) | 2,813 (56.6%) | 2,031 (64.2%) |
| 1 | 1,532 (24.7%) | 1,744 (30.1%) | 1,527 (30.7%) | 853 (26.9%) |
| *2+* | 709 (11.4%) | 859 (14.8%) | 627 (12.6%) | 282 (8.9%) |
| Mother |  |  |  |  |
| 0 | 4,661 (75.0%) | 3,870 (66.7%) | 3,091 (62.2%) | 2,120 (67.0%) |
| 1 | 1,205 (19.4%) | 1,457 (25.1%) | 1,384 (27.9%) | 766 (24.2%) |
| *2+* | 348 (5.6%) | 476 (8.2%) | 492 (9.9%) | 280 (8.8%) |
| **Psychological problems** | |  |  |  |
| *Father* |  |  |  |  |
| Yes | 5,913 (95.2%) | 5,562 (95.8%) | 4,798 (96.6%) | 3,123 (98.6%) |
| No | 301 (4.8%) | 241 (4.2%) | 169 (3.4%) | 43 (1.4%) |
| *Mother* |  |  |  |  |
| Yes | 5,591 (90.0%) | 5,279 (91.0%) | 4,632 (93.3%) | 3,019 (95.4%) |
| No | 623 (10.0%) | 524 (9.0%) | 335 (6.7%) | 147 (4.6%) |
| **Substance abuse** |  |  |  |  |
| *Father* |  |  |  |  |
| No | 5,738 (92.3%) | 5,421 (93.4%) | 4,715 (94.9%) | 3,080 (97.3%) |
| Yes | 476 (7.7%) | 382 (6.6%) | 252 (5.1%) | 86 (2.7%) |
| *Mother* |  |  |  |  |
| No | 6,036 (97.1%) | 5,713 (98.4%) | 4,913 (98.9%) | 3,152 (99.6%) |
| Yes | 178 (2.9%) | 90 (1.6%) | 54 (1.1%) | 14 (0.4%) |
